# Antifungal mode of action of macrocarpal C extracted from *Eucalyptus globulus* Labill (*Lan An*) towards the dermatophyte *Trichophyton mentagrophytes*

**DOI:** 10.1186/s13020-015-0068-3

**Published:** 2015-11-21

**Authors:** Jack Ho Wong, Kit-Man Lau, Yu-On Wu, Ling Cheng, Chun-Wai Wong, David Tai-Wai Yew, Ping-Chung Leung, Kwok-Pui Fung, Mamie Hui, Tzi-Bun Ng, Clara Bik-San Lau

**Affiliations:** School of Biomedical Sciences, The Chinese University of Hong Kong, Shatin, N.T., Hong Kong; Shenzhen Research Institute, The Chinese University of Hong Kong, Shatin, N.T., Hong Kong; Institute of Chinese Medicine, The Chinese University of Hong Kong, Shatin, N.T., Hong Kong; State Key Laboratory of Phytochemistry and Plant Resources in West China, The Chinese University of Hong Kong, Shatin, N.T., Hong Kong; Department of Microbiology, The Chinese University of Hong Kong, Shatin, N.T., Hong Kong

## Abstract

**Background:**

The fresh leaves of *Eucalyptus globulus* Labill. (*Lan An*) have been used in Chinese medicine for many years to treat dermatomycosis. Macrocarpal C was isolated from this herb and identified as its major antifungal component by bioassay-guided purification. This study aims to investigate the antifungal activity of macrocarpal C against *Trichophyton mentagrophytes*, which can cause tinea pedis.

**Methods:**

Fresh leaves of *E. globulus* were extracted with 95 % ethanol, and the resulting ethanolic extracts were dried before being partitioned with *n*-hexane. The *n*-hexane layer was then subjected to chromatographic purification to give macrocarpal C. The antifungal minimum inhibitory concentration (MIC) of macrocarpal C was determined using the standard M38-A2 method described by the Clinical Laboratory Standards Institute (CLSI). The mode of action of macrocarpal C was elucidated using three in vitro assays, including (1) a fungal membrane permeability test using SYTOX^®^ Green; (2) a reactive oxygen species (ROS) production test using 5-(and-6)-carboxy-2′,7′-dihydrodichlorofluorescein diacetate as a cell-permeable fluorogenic probe; and (3) a DNA fragmentation test based on terminal deoxynucleotidyl transferase dUTP nick-end labeling (TUNEL) detection. Terbinafine hydrochloride and nystatin were used as positive controls.

**Results:**

The suppression in the growth of *T. mentagrophytes* following its treatment with macrocarpal C was associated with an increase in the permeability of the fungal membrane (*P* = 0.0043 when compared to control); an increase in the production of intracellular ROS (*P* = 0.0063); and the induction of apoptosis as a consequence of DNA fragmentation (*P* = 0.0007).

**Conclusion:**

This study demonstrated that the antifungal action of macrocarpal C was associated with increases of membrane permeability, intracellular ROS and DNA fragmentation.

## Background

Tinea pedis is a dermatophyte infection of the feet which is mainly caused by *Trichophyton mentagrophytes* and *Trichophyton rubrum* [1]. This particular infection is one of the most commonly diagnosed skin infections throughout the different countries of the world [1–5]. Although tinea pedis is not life-threatening, it can cause stress, pain and discomfort to those suffering from the disease, and consequently has a significant impact on the quality of life and well-being of these patients [3, 6]. Although several medicines, including griseofulvin, itraconazole, fluconazole and terbinafine, are commercially available for the treatment of tinea pedis, their applications have been limited in some cases by narrow spectrums of activity, drug resistance, toxicity or the occurrence of unwanted drug–drug interactions [7–9]. The development of new antifungal agents with improved properties is therefore necessary to explore the underlying mechanisms of action of these fungi and provide deeper insights to facilitate future drug development programs.

In our previous study, we isolated macrocarpal C from the fresh leaves of *Eucalyptus globulus* Labill. (Lan An) and demonstrated its antifungal activity [10]. Macrocarpal C inhibited the growth of three different dermatophytes, including *T. mentagrophytes*, *T. rubrum* and *Paecilomyces variotii*. This study aims to elucidate the mode of action of macrocarpal C by investigating its impact on three key processes, including (1) fungal membrane permeability; (2) the production of reactive oxygen species (ROS); and (3) the induction of apoptosis by DNA fragmentation.

## Methods

### Raw herb

Eucalypti Globuli Folium was collected in Cao Pu Zhen, Anning City, Kunming, Yunnan Province, China and authenticated by Dr. Gao Li of the Yunnan Institute of Materia Medica according to the Flora Reipublicae Popularis Sinicae [11]. Herbarium voucher specimens were deposited at the museums of Yunnan Institute of Materia Medica, Yunnan Province, China and the Institute of Chinese Medicine, The Chinese University of Hong Kong with the voucher specimen numbers 20100074 and 2012-3358, respectively.

### Chemicals

All of the chemicals used in the current study were purchased as the reagent grade from Sigma-Aldrich (St. Louis, MO, USA). An In Situ Cell Death Detection Kit was purchased from Roche Diagnostics NZ Ltd (Mt. Wellington, Auckland, New Zealand). SYTOX^®^ Green and LIVE/DEAD^®^ yeast viability kits were purchased from Life Technologies Corporation (Carlsbad, CA, USA).

### Fungal strain

*T.mentagrophytes* ATCC 9129 (ATCC, Manassas, VA, USA) was used throughout this study. This material was cultured at 25 °C on modified Sabouraud dextrose agar (MSDA) slants containing mycological peptone 10 g/L, glucose 40 g/L and agar 15 g/L at a pH in the range of 5.4–5.8.

### Isolation of macrocarpal C

Fresh *E. globulus* leaves were extracted two times for 1 h with 95 % ethanol under reflux conditions. The resulting ethanolic extracts were combined, dried and partitioned with *n*-hexane. This procedure was repeated one more time, and the resulting *n*-hexane fractions were combined and subjected to chromatographic purification for the isolation of macrocarpal C, as previously described [10]. The purity of the macrocarpal C obtained in this way was higher than 95 %, as determined by HPLC.

### In vitro antifungal susceptibility test

#### Cultures of the dermatophyte

Pure cultures of the dermatophytes were produced by aerobically culturing the fungi at 35 °C for 1 week on modified Sabouraud dextrose agar (MSDA) slants. A 3-mL portion of saline was added to the slants and the subsequent probing of the colonies with a sterile cotton-tipped swab resulted in the formation of a suspension, which was filtered twice through sterile gauze to eliminate hyphal filaments. The final inoculum of conidia was acquired by the dilution of the mixture with RPMI-1640 (buffered with MOPS) medium to 1–3 × 10^3^ CFU/mL.

#### Determination of the minimum inhibitory concentration (MIC)

The MIC was defined as the lowest concentration at which the growth of the microorganism was completely inhibited. The M38-A2 broth dilution method was used in the current study according to a slightly modified version of the procedure described by the Clinical Laboratory Standards Institute (CLSI) [12]. Macrocarpal C was diluted with RPMI-1640 to achieve concentrations ranging from 500 to 0.06 mg/L in the U-shaped wells of a 96-well microtiter plate. Equal volumes of drug solution and microbial suspension were mixed to make up a final volume of 200 µL. Growth control wells containing RPMI-1640 and microorganisms on each microtiter plate were used for quality control. Sterility control wells containing drug and RPMI but no microorganism were also included in each plate. The plates were then wrapped with paraffin to prevent losses due to evaporation and incubated at 35 °C. The endpoints were visually read every 24 h with a reading mirror and compared with the growth control for up to 4 days. All of these experiments were independently performed in duplicates thrice.

### In vitro mechanistic studies

#### Effects on the membrane permeability

This assay was carried out by examining the uptake of SYTOX^®^ Green, which is a high-affinity nuclear stain capable of entering cells with compromised membranes [13]. Fungal cells were incubated in the presence of macrocarpal C, terbinafine hydrochloride, nystatin (positive control) or phosphate buffer saline (negative control) at 37 °C for 24 h. At the end of the incubation, SYTOX^®^ Green was added to the fungal cultures at 0.5 µM (final concentration). After 10 min, the fluorescence emitted by the fungal cells was measured with a fluorescence spectrometer (SpectraMax i3 Multi-Mode Detection Platform, Molecular Devices, LLC, Sunnyvale, CA, USA) at an excitation wavelength of 488 nm and an emission wavelength of 540 nm.

#### Effect on the production of reactive oxygen species (ROS)

The cell-permeable and fluorogenic probe 5-(and-6)-carboxy-2′,7′-dihydrodichlorofluorescein diacetate (carboxy-H_2_DCFDA) was used to determine ROS production in the fungal cells [13]. The fungal cells were cultured for 3 h in the presence or absence of macrocarpal C. The medium was then replaced with PBS. After incubation with 25 µM carboxy-H_2_DCFDA in PBS at 37 °C for 30 min, the cells were washed twice with fresh pre-warmed PBS. The fluorescence emitted by the fungal cells was quantified with a fluorescence spectrometer at an excitation wavelength of 488 nm and an emission wavelength of 540 nm.

#### Effects on DNA fragmentation

A TUNEL assay was conducted to detect the occurrence of any DNA fragmentation [14]. Briefly, *T.mentagrophytes* cells were incubated with macrocarpal C at 1 × MIC for 3 h, washed twice with PBS and fixed with a fixative solution of 4 % paraformaldehyde in PBS (pH 7.4) for 1 h at 20 °C. The cells were then rinsed twice with PBS and incubated on ice for 2 min with the permeabilization solution described above. The cells were subsequently rinsed with PBS and labeled using an In Situ Cell Death Detection Kit according to the manufacturer’s instructions. Briefly, a 50 µL sample of the TUNEL reaction mixture was added to the cells, and the resulting mixture was incubated at 37 °C for 60 min in the dark. The cells were then rinsed with PBS and examined using a fluorescence spectrometer.

### Statistical analyses

All of the data in this study have been presented as the mean values ± standard deviations (SD) and analyzed using Student’s *t* test. The differences between control and test groups were considered statistically significant for *P* < 0.05. Multiple comparisons among groups were conducted using one-way analysis of variance (ANOVA) followed by post hoc Tukey test, with significance level α set at 0.05. Dose-dependent changes were determined by visually inspecting the graphs.

## Results and discussion

The fresh leaves of *E. globulus* are used in Chinese medicine for the treatment of dermatomycosis, cough, headache, influenza and eczema. Furthermore, the methanol-dichloromethane (1:1–v/v) extract of the fresh leaves of *E. globulus* inhibited *T. mentagrophytes* with an MIC value of 31 µg/mL [15]. In our previous study, macrocarpal C was isolated from *E. globulus* leaves using a bioassay-guided fractionation method [10]. This material was subsequently shown to be the active antifungal component of these leaves and inhibited the growth of dermatophytes in vitro with an MIC value of 1.95 µg/mL towards *T. mentagrophytes*.

**Fig. 1:**
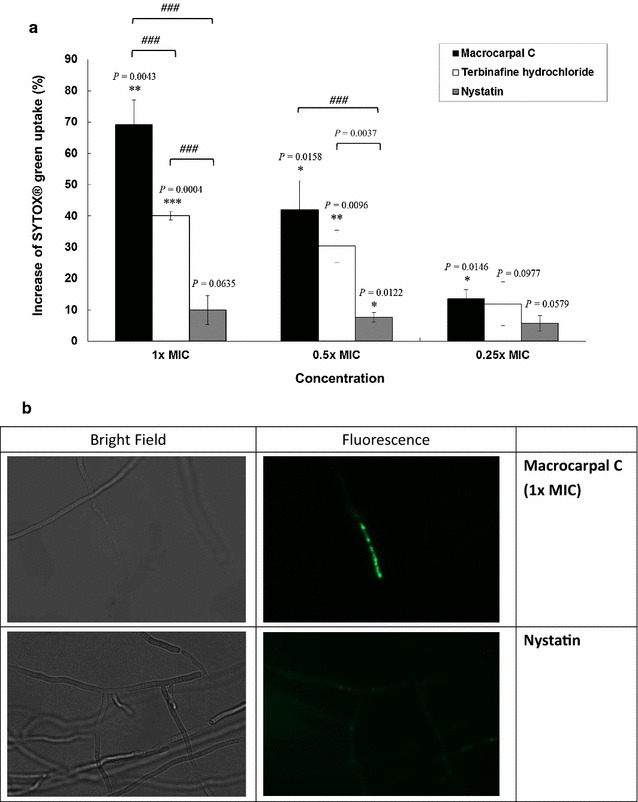
**a** Effect of macrocarpal C on SYTOX^®^ Green uptake into *Trichophyton mentagrophytes* after 24 h of treatment. The MIC values of macrocarpal C, terbinafine hydrochloride and nystatin were determined to be 1.95, 0.625 and 1.25 µg/mL, respectively. Data are presented as the mean values ± SD (n = 3) (Three independent experiments), with *P < 0.05, **P < 0.01 and ***P < 0.001 when compared with the control (0 %) using Student’s *t*-test. ^*###*^ *P* < 0.001 for multiple comparison among groups using one-way analysis of variance (ANOVA) followed by post hoc Tukey test. **b** Pictures taken under a fluorescence microscope (Nikon Ti-E)

Macrocarpal C, the well-known antifungal drug terbinafine hydrochloride and the positive control nystatin all led to a dose-dependent increase in the uptake of SYTOX^®^ Green into the fungal cells. Macrocarpal C led to a 69.2 % increase in the uptake of SYTOX^®^ Green at a concentration of 1 × MIC (*P* = 0.0043); a 42.0 % increase at a concentration of 0.5 × MIC (*P* = 0.0158); and a 13.6 % increase at a concentration of 0.25 × MIC (*P* = 0.0146) compared with the negative control (Fig. 1). Macrocarpal C was found to be more potent than terbinafine hydrochloride and nystatin at the same MIC. These results therefore indicated that macrocarpal C could be inhibiting the growth of the fungal cells by altering their membrane permeability.

**Fig. 2:**
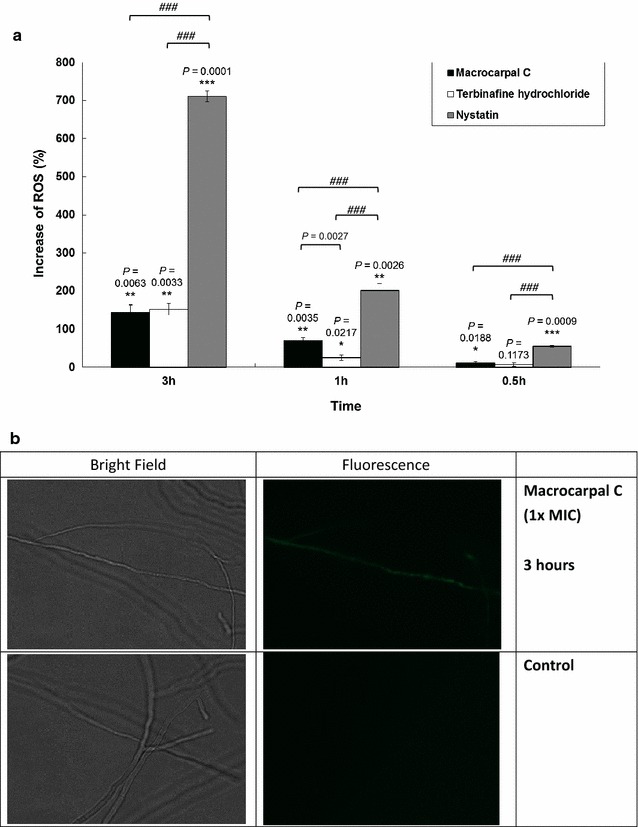
**a** Effect of macrocarpal C on ROS release from *Trichophyton mentagrophytes* after treatment at 1 × MIC for different durations. The MIC values of macrocarpal C, terbinafine hydrochloride and nystatin were determined to be 1.95, 0.625 and 1.25 µg/mL, respectively. Data are presented as the mean values ± SD (n = 3) (Three independent experiments), with *P < 0.05, **P < 0.01 and ***P < 0.001 when compared with the control (0 %) using Student’s *t*-test. ^*###*^ *P* < 0.001 for multiple comparison among groups using one-way analysis of variance (ANOVA) followed by post hoc Tukey test. **b** Pictures taken under a fluorescence microscope (Nikon Ti-E)

Macrocarpal C and terbinafine hydrochloride led to only slight increases in ROS production at 0.5 h. However, these increases became much more significant as the duration of the incubation was extended to 1 h and then 3 h (11.6 %, *P* = 0.0188 at 0.5 h; 70.1 %, *P* = 0.0035 at 1 h and 144.3 %, *P* = 0.0063 at 3 h for macrocarpal C compared with the control). The positive control nystatin led to much greater increases in ROS production (Fig. 2).

**Fig. 3:**
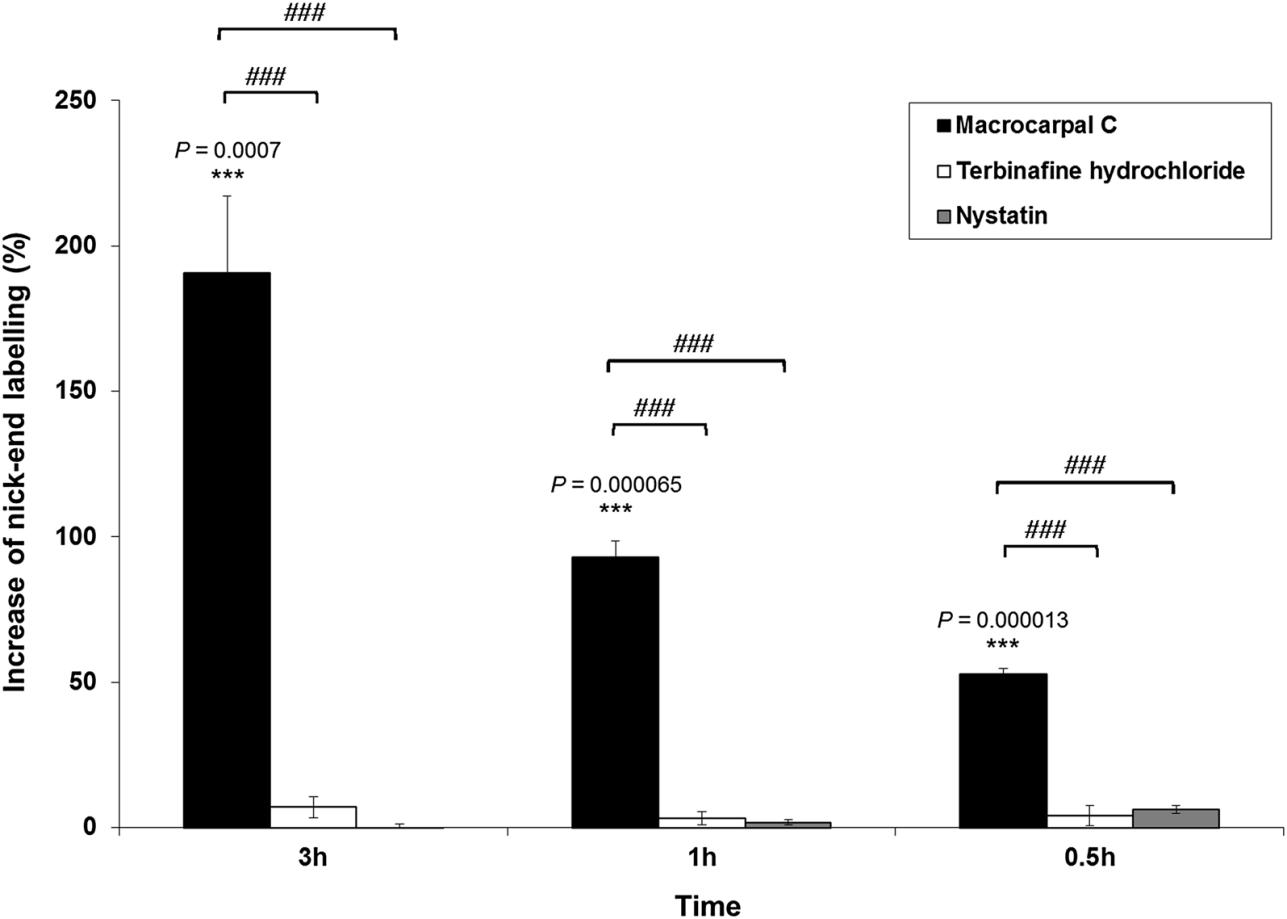
Effect of macrocarpal C on the nick-end labeling of *Trichophyton mentagrophytes* after treatment at 1 × MIC for different durations. The MIC values of macrocarpal C, terbinafine hydrochloride and nystatin were determined to be 1.95, 0.625 and 1.25 µg/mL, respectively. Data are presented as the mean values ± SD (n = 3) (Three independent experiments), with ***P < 0.001 when compared with the control (0 %) using Student’s *t*-test. ^*###*^ *P* < 0.001 for multiple comparison among groups using one-way analysis of variance (ANOVA) followed by post hoc Tukey test

The results of the TUNEL assay revealed that macrocarpal C led to a pronounced, time-dependent increase in the level of nick-end labeling compared with the control (all *P* < 0.001) (Fig. 3). Macrocarpal C could therefore induce DNA fragmentation in fungal cells.

To the best of our knowledge, there have been no studies pertaining to the use of SYTOX^®^ Green in conjunction with a TUNEL assay for *Trichophyton* spp. ROS are involved in the fungicidal activity of antimicrobial photodynamic inhibitors towards *T. rubrum* [16]. Furthermore, antifungal azoles have been reported to have a significant impact on the membrane permeability in *Trichophyton* spp. [17, 18].

Macrocarpal C led to an increase in nick-end labeling 0.5 h after the treatment of the fungal cells. Furthermore, this increase in nick-end labeling continued with time. This result indicated that macrocarpal C was inducing the fragmentation of the DNA in the dermatophytic fungus. It is noteworthy that nystatin and terbinafine hydrochloride both failed to mimic this action when they were tested at the same MIC. Macrocarpal C stimulated SYTOX^®^ Green uptake and augmented ROS generation. The former of these two results indicated that the treatment of the fungal cells with macrocarpal C led to an increase in the permeability of the dermatophyte membrane, which allowed the nuclear stain SYTOX^®^ Green to enter the fungal cells through the compromised membrane. The treatment of the cells with terbinafine hydrochloride also led to an increase in their permeability. In contrast, nystatin had no discernible impact on the permeability of the fungal cells. Macrocarpal C triggered an increase in the ROS in the fungal cells, albeit to a lesser extent than the positive control nystatin. Several antifungal agents have been reported to stimulate the production of ROS, which subsequently caused damage to the fungal cells [19, 20].

There has been a significant increase in the number of biological studies conducted during the last decade concerning the extracts of traditional Chinese medicinal herbs. Most notably, these studies have culminated in the isolation of a large number of interesting compounds with a wide range of biological effects. However, research concerning herbal medicines must be standardized to ensure that the studies performed by one researcher can be readily repeated by another researcher. In this study, we used standard methods (e.g., the antimicrobial susceptibility test to determine the MIC value of macrocarpal C) because we wanted to make it as easy as possible for all researchers to follow up on our findings.

## Conclusion

This study demonstrated that the antifungal action of macrocarpal C was associated with increases of membrane permeability, intracellular ROS and DNA fragmentation.

## Abbreviations

MIC: minimum inhibitory concentration
ROS: reactive oxygen species
TUNEL: terminal deoxynucleotidyl transferase dUTP nick-end labeling
